# Simultaneous HPLC Determination of Butenafine Hydrochloride and Betamethasone in a Cream Formulation

**DOI:** 10.4103/0250-474X.58194

**Published:** 2009

**Authors:** R. Ankam, K. Mukkanti, S. Durgaprasad, M. Khan

**Affiliations:** Glenmark Pharmaceuticals Ltd, R&D Centre, Plot No. C-152, MIDC, Malegaon, Sinnar-422 113, India; 1 Centre for Environment IPGSR, JNT University, Hyderabad-500 028, India; 2 Indian Immunologicals Ltd, Rakshapuram, Gachibowli, Hyderabad-500 019, India

**Keywords:** RP-HPLC method development and validation, simultaneous determination, butenafine hydrochloride, betamethasone

## Abstract

A fast, specific, accurate and precise reverse phase high performance liquid chromatographic method was developed for the simultaneous determination of butenafine hydrochloride and betamethasone in cream formulation. The determination was carried out on licrocart licrosphere RP-select B (250×4.6 mm, 5 μ) column in isocratic mode, the mobile phase consisting of 50 mM ammonium acetate buffer and acetonitrile in the ratio of 60:40, adjusted to pH 4.5 ± 0.1 with glacial acetic acid. The flow rate was 2.0 ml/min and eluent was monitored at 254 nm. The retention times of butenafine hydrochloride and betamethasone were 4.70 min and 7.76 min, respectively, and the resolution factor was greater than 4.0. Linearity of butenafine hydrochloride and betamethasone were in the range of 100-300 μg/ml and 5-15 μg/ml, respectively. The proposed method is also found to be precise and robust for the simultaneous determination of butenafine hydrochloride and betamethasone in cream formulation.

Earlier days the practices of curing the skin diseases with single drug molecule for prolong period, this general practice leads to develop drug resistance for bacterial and fungal strains. Now the practice has been changed and combination of drug molecules implies for simultaneous mechanism of action, this leads to application of drugs for short period, which will exhibit fast curing and minimum development of drug resistance to the microbial strains. There is a simultaneous requirement of fast, potential analytical methods to determine the drugs in the given formulation.

The past decade has witnessed a significant increase in the prevalence of resistance to antibacterial and antifungal agents. Resistance to antimicrobial agents has important implications for morbidity, mortality and health care. Antifungal can be grouped into three classes based on their site of action[1]. Azoles, which inhibit the synthesis of ergosterol (the main fungal sterol); polyenes, which interact with fungal membrane sterols physic chemically and 5-fluorocytosine, which inhibits macromolecular synthesis. Many different types of mechanisms contribute to the development of resistance to antifungal agents. These mechanisms include alteration in drug target, alteration in sterol biosynthesis, reduction in the intercellular concentration of target enzyme, and over expression of the antifungal drug target.

Butenafine hydrochloride (n-4-ter-butyl-benzyl-N-methyl-1-naphthaline methylamine hydrochloride) is a new benzylamine derivative with a chemical structure and mode of action similar to allylamine antifungal. It has the empirical formula C_23_H_27_ N.HCl and a molecular weight of 353.93. It inhibits squalene epoxidase, an enzyme which converts squalene to lanosterol and leads to accumulation of squalene and is primarily fungicidal against dermatophytes. The azoles antifungal have been extensively used to treat dermatophytosis since the 1970s. The most commonly used topical agents are clotrimazole and miconazole. The azoles act as fungi-static agents by inhibiting the cytochrome P450-dependent enzyme lanosterol 14-demethylase, which is important for the biosynthesis of ergosterol, a component of fungal cell membrane. It is freely soluble in methanol, ethanol and chloroform and slightly soluble in water.

Betamethasone dipropionate is designated chemically as 9-fluoro-11b-hydroxy-16b-methyl-3,20 dioxopregna-1,4-diene-17,21-diyl dipropionate. It has the empirical formula C_28_H_37_FO_7_ and a molecular weight of 504.6. Betamethasone dipropionate is a synthetic adrenocorticosteroid, for dermatologic use. Betamethasone, an analog of prednisolone has high corticosteroid activity and slight mineralocorticoid activity. Betamethasone dipropionate is a white or almost white, crystalline powder, practically insoluble in water, freely soluble in acetone and in methylene chloride, sparingly soluble in alcohol.

The developed cream formulation contained 1% butenafine hydrochloride synthetic antifungal agent and 0.05% synthetic corticosteroid betamethasone as betamethasone dipropionate is member of the class of steroids. A survey of literature revealed that few chromatographic and spectrophotometric methods are reported for determination of butenafine hydrochloride and betamethasone individually[2–7]. However there is no HPLC method reported for simultaneous determination of butenafine hydrochloride and betamethasone from combine dosage form. The present investigation describes a fast, precise and accurate reverse phase HPLC method for simultaneous estimation of butenafine hydrochloride and betamethasone in the cream formulation.

The drug sample, butenafine hydrochloride was procured from Hetero Drugs Limited, Hyderabad, India, betamethasone dipropionate and cream sample were used in-house. HPLC grade acetonitrile, ammonium acetate and glacial acetic acid were purchased from Merck India Limited, Mumbai, India.

An isocratic high performance liquid chromatography (Jasco system) with intelligent sampler (AS-2057), quaternary gradient pump (PU-2080), intelligent UV/Vis detector (UV-2075), intelligent column oven (CO-2065) was used. The chromatography column used was licrocart licrosphere RP-select B (250×4.6 mm i.d., particle size 5 μ) column.

A mixture of 50 mM ammonium acetate buffer (adjusted pH to 4.5 with ten percent solution of acetic acid) and acetonitrile in the ratio 60:40 v/v was filtered through 0.45 μ membrane filter and used as mobile phase. The flow rate of mobile phase was maintained at 2.0 ml/min. For calibration, standard butenafine hydrochloride and betamethasone solutions were prepared at concentration range of 100-300 μg/ml for butenafine hydrochloride and 5-15 μg/ml for betamethasone in mobile phase. These standard solutions were injected in duplicate and the average detector response measured at 254 nm.

In-house research and development samples (Lot-1, 2 and 3) two tubes each 5 g cream sample was taken in 100 ml beaker and mixed properly. An accurately weighed quantity of cream equivalent to 20 mg of butenafine hydrochloride and 1 mg of betamethasone was taken in 100 ml volumetric flask and dissolved in mobile phase. Volume was made up to mark with mobile phase. Whole solution was transferred in 250 ml volumetric flask and chilled it in ice bath for 10 min. The solution was filtered through Whatman filter paper No. 40. The aliquot portion of the filtrate was further diluted to get final concentration and detection was done at 254 nm. The results obtained in the experiment were tabulated in Table 1.

**TABLE 1:** ANALYSIS OF CREAM CONTAINING BUTENAFINE HCL AND BETAMETHASONE

Formulation	Label Content % w/w	Amount found % w/w	% Drug found	Standard Deviation
Butenafine HCl				
Lot-1	1.0	10.004	100.40	0.59
Lot-2	1.0	0.999	99.93	0.55
Lot-3	1.0	0.989	98.93	0.67
Betamethasone				
Lot-1	0.05	0.050665	101.33	0.60
Lot-2	0.05	0.049665	99.33	0.59
Lot-3	0.05	0.49165	98.33	0.69

All lots are prepared in in-house facility and values are the average of six determinations (n=6)

The method was validated for accuracy, precision, specificity and robustness (Table 2). The accuracy of the method was determined by calculating recoveries of butenafine hydrochloride and betamethasone by placebo spiked recovery. Known amounts of butenafine hydrochloride (100, 200 and 300 μg/ml) and betamethasone (5, 10 and 20 μg/ml) were added to placebo preparation and the amount of butenafine hydrochloride and betamethasone were estimated by measuring the peak areas.

**TABLE 2:** RESULTS OF METHOD VALIDATION EXPERIMENTS OF BUTENAFINE HCL AND BETAMETHASONE

Performance parameters	Butenafine HCl	Betamethasone
Linearity and range	100-300 μg/ml	5-15 μg/ml
Regression coefficient (r^2^)	0.9998	0.9998
Regression equation (y=m×+c)*		
Accuracy	1.94	1.99
Precision (RSD, n=6)	1.17	1.69
Specificity	Interference not observed	Interference not observed
Stability analytical solution	0, 6, 12, 18, 24h. (n=5)	0, 6, 12, 18, 24h. (n=5)
(normal conditions)	%RSD 1.10	%RSD 1.89
Stability of analytical solution	0, 6, 12, 18, 24h. (n=5)	0, 6, 12, 18, 24h. (n=5)
(In dark refrigerator)	%RSD 1.13	%RSD 1.9
Intermediate precision (RSD)	0.81	0.66

RSD = Relative Standard Deviation, y = peak area, x = concentration in μg/ml, m = slope, c = intercept

Linearity experiment was performed by using definite concentrations of butenafine hydrochloride (100, 150, 200, 250 and 300 μg/ml) and betamethasone (5, 7.5, 10, 12.5 and 15 μg/ml) solutions were injected and peak areas were recorded. A linear graph was plotted by using the peak areas against concentration in μg/ml.

The instrument precision was evaluated by injecting the butenafine hydrochloride (200 μg/ml) and betamethasone (10 μg/ml) solution six times repeatedly and peak areas were measured. The results are reported in terms of relative standard deviation. The intra-day and inter-day precision study of butenafine hydrochloride and betamethasone was carried out by estimating the corresponding responses six times on the same day and six times on the second day. The results obtained were reported in terms of relative standard deviation (RSD). The specificity was estimated by spiking known quantity of drug in to placebo preparation. The chromatography was performed by using appropriate dilutions and quantities of drugs were estimated. Robustness of the method was studied by deliberately changing the experimental conditions like flow rate, percentage of organic phase. Stability of the standard and sample solutions were observed at 25+2° for 24 h. The sample solution was assayed at every 6 h intervals up to 24 h.

Optimization of mobile phase was performed based on resolution, total runtime, asymmetric factor and theoretical plates obtained for butenafine hydrochloride and betamethasone. Mobile phase consisting of 50 mM ammonium acetate buffer and acetonitrile in the ratio of 60:40, adjusted pH 4.5±0.1 with glacial acetic acid was selected which gave sharp, well resolved peaks for butenafine hydrochloride and betamethasone (fig. 1). The retention time for butenafine hydrochloride and betamethasone were 4.7 and 7.7 min, respectively. The asymmetry factor for butenafine hydrochloride and betamethasone were 1.3, 1.1, respectively. UV spectra of butenafine hydrochloride and betamethasone showed that both the drugs absorbed appreciably at 254 nm, so the same was selected as the detection wave length during the studies. The linearity curve was found to be linear over the range of 100-300 μg/ml for butenafine hydrochloride and 5-15 μg/ml for betamethasone. The data of regression analysis of the calibration curve are shown in Tables 3 and 4.

**Fig. 1:**
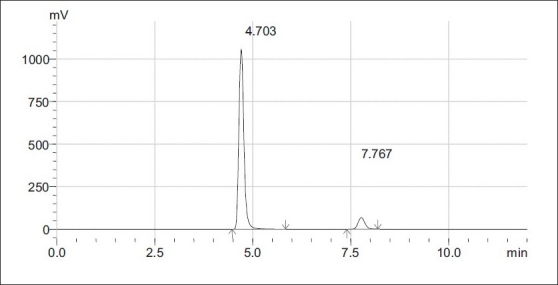
Typical chromatogram of butenafine HCl and betamethasone. Liquid chromatogram showing well resolved peaks of butenafine HCl (200 μg/ml, R_t_ = 4.07) and betamethasone (10 μg/ml, R_t_ = 7.76) at λ 254 nm.

**TABLE 3:** RESULTS FOR REGRESSION ANALYSIS DATA OF BUTENAFINE HCL

Linearity Level	Conc. (ppm)	Experimental area (a)	Predicted area (y) y=mx+c	Residuals(b) b= a-y
Level-50%	101.87	6118269	6094915.2	23353.8
Level-75%	152.80	9141247	9064783.1	76463.9
Level-100%	203.74	11855406	12034651	-179245
Level-125%	254.67	15040202	15004518.9	35683.1
Level-150%	305.60	18018131	17974386.8	43744.2

The correlation coefficient is 0.9998, intercept (c) is 155179.40 and slope (m) is 58308.31

**TABLE 4:** RESULTS FOR REGRESSION ANALYSIS DATA OF BETAMETHASONE

Linearity Level	Conc. (ppm)	Experimental area (a)	Predicted area (y) y=mx+c	Residuals(b) b= a-y
Level-50%	5.01	929154	936553	-7399
Level-75%	7.52	1415671	1396485.3	19185.7
Level-100%	10.02	1836002	1856417.6	-20415.6
Level-125%	12.53	2329220	2316349.9	12870.1
Level-150%	15.04	2772041	2776282.2	-4241.2

The correlation coefficient is 0.9998, intercept (c) is 16688.40 and slope (m) is 183532.44

The accuracy of the method was determined by calculating recoveries of butenafine hydrochloride and betamethasone by placebo spiked method. The recoveries obtained were 99.26-101.98 % for butenafine hydrochloride and 98.02-101.88 % for betamethasone. The high values indicate that the method is accurate. Instrument precision was determined by performing injection repeatability test for standard solution, and the RSD values for butenafine hydrochloride and betamethasone were found to be 1.94 and 1.99, respectively.

For inter-day study RSD values were found to be 0.81, 0.66 for butenafine hydrochloride and betamethasone respectively. The low RSD values indicate that the method is precise. Robustness of the method was studied by changing the flow rate of the mobile phase from 2.0 ml/min to 1.8 ml/min and 2.2 ml/min. Using 1.8 ml/min flow rate, retention time for butenafine hydrochloride and betamethasone were observed to be 5.2 and 8.5 min, respectively and with 2.2 ml/min flow rate retention time for butenafine hydrochloride and betamethasone were observed to be 4.2 and 6.9 min, respectively without affecting the resolution of the drugs. When mobile phase composition was changed to increase in 2% organic phase, the retention time for butenafine hydrochloride and betamethasone were observed to be 3.9 and 5.9 min, respectively and decrease in 2% organic phase, the retention time for butenafine hydrochloride and betamethasone were observed to be 5.8 and 8.2 min, respectively. The solution stability study revealed that butenafine hydrochloride and betamethasone solutions were stable for 24 h without detectable degradation and the percentage recovery of both the drugs were found to be more than 98.02%.
